# Exploring different interventions for Relative Energy Deficiency in Sport (REDs): A systematic review^☆^

**DOI:** 10.1016/j.jsampl.2024.100085

**Published:** 2025-01-02

**Authors:** Rosie Rudin, Louisa Harris, Hollie White, Lucy Hammond

**Affiliations:** Warwick Medical School, University of Warwick, Coventry CV4 7AL, UK

**Keywords:** Female athlete triad, Low energy availability, Management, Treatment, Athlete

## Abstract

**Objectives and design:**

Relative Energy Deficiency in Sport (REDs) is caused by an imbalance in energy intake and expenditure through exercise, which leads to low energy availability. Although awareness of REDs is improving, a synthesis into the available literature to determine the efficacy of different interventions in athletes with REDs is needed. Subsequently, this may inform clinicians and athletes of the most appropriate interventions.

**Methods:**

Medline, Embase and Web of Science databases were searched using keywords and their combinations. Two independent reviewers screened the retrieved studies by title, abstract and full text. The Joanna Briggs Institute critical appraisal tool was used to assess risk of bias. Data were extracted into an excel spreadsheet and a narrative synthesis performed.

**Results:**

The search retrieved 982 papers, and after screening, 11 were eligible for inclusion, including increase in energy intake (n ​= ​3), increase in energy intake and decrease in energy expenditure (n ​= ​1), dietary approaches (n ​= ​1), education and counselling (n ​= ​5) and hormonal intervention (n ​= ​1). Increasing energy intake enabled return of menses, yet did not significantly increase bone mineral density. Dietary approaches significantly improved body image, tension, vigour and depression. Nutrition education improved bone health, energy availability, and eating behaviours in athletes, while hormonal interventions had limited effects on bone mineral density.

**Conclusion:**

Increasing energy intake is essential in the management of REDs and may be more effective when used alongside education and hormonal treatment. Future research is needed to allow better analysis of the efficacy of interventions due to the limited research available.

## 1 Introduction

Relative Energy Deficiency in Sport (REDs) is the outcome of chronic (weeks to months) or acutely severe (days) low energy availability (LEA) [1]. Energy availability (EA) is the difference between energy intake and energy expenditure, and insufficient EA in the sporting context can lead to LEA [2]. The mathematical formula for EA, which determines the amount of energy the body can allocate to functions essential to daily living, is EA ​= ​[Energy intake (kcal) – Exercise Energy Expenditure (kcal)]/Fat Free Mass (kg/day) [1]. The threshold for LEA in females is 30 ​kcal/kg Fat Free Mass/day, and in males is ∼9 to 25 ​kcal/kg Fat Free Mass/day [1]. The variability in these numbers shows a consensus for this cut off has not yet been reached [1]. LEA causes physiological and psychological changes which can lead to a decline in sports performance, such as decreased strength and endurance [2]. Changes may be short-term and have little impact on long-term health - termed adaptable LEA, or can be long-term and significant - termed problematic LEA [1]. The relationship between menstrual dysfunction, disordered eating and reduced bone loss was originally characterised as the Female Athlete Triad, as it was thought to only affect women [3]. However, there is increasing evidence which implicates REDs in female and male athletes from any sporting discipline and at any performance level. Thus, the International Olympic Committee (IOC) altered the name in 2014 [3]. Despite the increasing knowledge of REDs, many doctors are still unaware of the meaning of REDs, let alone interventions for this condition. A survey [4] showed that only 36​% of physicians had knowledge of REDs and only 13​% of healthcare professionals reported they felt comfortable to treat the condition. Nevertheless, a recent 2023 IOC consensus has provided evidence that this gap in knowledge is narrowing [1]. Best practices for treating REDs were mentioned, yet they also highlighted a clear need for more research into the efficacy of treatments [1].

The prevalence of REDs remains unknown due to ambiguity in diagnosis and differences in methods to determine EA. Athletes are at a higher risk of developing REDs if they experience disordered eating, or participate in endurance sports, or rely on aesthetics, for example gymnastics, compared to lower risk ball game sports, such as softball and basketball [2,5]. Many aspects of health are negatively impacted by LEA, including menstrual dysfunction, reduced testosterone levels in males, decreased immune response, reduced metabolism, increased risk of injury and illness, reduced bone turnover, altered sleep, delayed growth and altered cardiovascular and gastrointestinal functioning [1,2,6]. REDs leads to a stagnation or deterioration in athletic performance through the downregulation of hormones. For example, in female swimmers with LEA, ovarian, triiodothyronine, and Insulin-like Growth Factor 1 hormones were downregulated, and these athletes were unable to improve their 400 ​m swimming time, in comparison to their counterparts who had adequate hormone levels and EA [7].

Measuring EA is difficult; instead, ascertaining hormone levels allows for a more sensitive measurement into the risks of REDs and is usually used following screening questionnaires in athletes [8]. Despite this, REDs is ultimately a diagnosis of exclusion, with the REDs clinical assessment tool used to stratify athletes according to a traffic light system based on clinical features [9]. In practice, a range of interventions are used to improve symptoms of REDs. Primarily, this can be achieved through increasing energy intake and decreasing energy expenditure, and may involve dieticians, physicians, coaches, physiologists and psychologists [1,9]. Educating athletes and support staff on optimal nutritional and exercise programmes is also important [1,10]. Nutritional supplements, such as calcium and vitamin D, may be advised [11]. Despite awareness of REDs increasing as research into this important topic continues, previous reviews have focused solely on the effects of nutritional interventions on menstrual dysfunction or non-pharmacological interventions for REDs, stating that there are no pharmacological agents to treat REDs [11,12]. However, hormonal transdermal oestradiol therapy can be used in those with REDs and may be combined with oral contraceptives (OC), such as progesterone, and so a synthesis of research into the efficacy across a range of available interventions is lacking [13,14]. Therefore, to enable clinicians and athletes to make more informed choices on a wider variety of evidence-based interventions, the aim of this study was to synthesise the available literature to determine the efficacy of different interventions in athletes with REDs.

## 2 Methods

### 2.1 Study protocol registration and reporting

This systematic review aimed to determine the efficacy of different interventions in athletes with REDs. The efficacy of different interventions for REDs was measured using a range of proxy measurements, including physiological outcomes, such as menstrual function, bone health, metabolic function, as well as the psychological impact and athletic performance. This systematic review followed the guidelines specified in the Preferred Reporting Items for Systematic Review and Meta-Analyses and this was independently reviewed [15]. The study protocol has been registered with PROSPERO, an international database of prospectively registered systematic reviews in health and social care (CRD42023474552).

### 2.2 Search strategy

Medline, Embase and Web of Science databases were used to search for appropriate published articles. The full search strategy is found in the supplemental files. Keywords and their combinations used for the search were: (REDs OR Female Athlete Triad OR Low Energy Availability) AND athlet∗ AND (Treatment OR Management OR Intervention). Boolean operators such as ‘AND’ and ‘OR’ were used to support with this search process. The ‘AND’ limited the quantity of papers shown in the search. The ‘OR’ broadened the search, which is essential as the term REDs was formally described as the Female Athlete Triad and LEA. Truncations were used, shown by the symbol ‘∗’, to allow for different variations of words. The search process was reviewed with the university librarian. The searches were imported into Rayyan to remove duplicate papers [16].

### 2.3 Inclusion and exclusion criteria

#### 2.3.1 Inclusion criteria

The planned inclusion criteria presented in the protocol were further clarified with specific examples. Studies were included if they reported on athletes who a) were stated as being high risk of REDs in original research articles or exhibited one or more symptoms of REDs (for example LEA, menstrual dysfunction or disorder eating etc.) b) were from any sporting discipline at any performance level c) were over the age of 16 years d) designed as randomised controlled trials, quasi-experimental studies, cohort studies or case control studies.

#### 2.3.2 Exclusion criteria

Non-human studies were excluded, alongside consensus and conference statements, reviews, opinions, editorials, thesis, dissertations, and case reports. Studies not in the English language were also excluded.

### 2.4 Screening

The titles and abstracts were screened by two independent reviewers in Rayyan [16]. Studies selected for a full text review were imported into Covidence for screening [17]. In case of discrepancies on inclusion, these were discussed and resolved.

### 2.5 Critical appraisal

Two reviewers independently assessed the quality of studies that met the eligibility criteria using the appropriate Joanna Briggs Institute critical appraisal tool [[18], [19], [20]]. Where there were discrepancies on the risk of bias, these were discussed and resolved.

### 2.6 Data extraction

Data were extracted into an excel spreadsheet (shown in supplemental files) by two reviewers, using a reviewer guidance form (shown in supplemental files). The data included in this extraction comprised authors, year of publication, title of article, geographical location, study design, participant information (number, age, sex, sporting discipline, symptoms), number of participants who did not complete the study, intervention, duration, and outcomes. Moreover, EndNote was used to collate references [21].

### 2.7 Synthesis of extracted data

Owing to the heterogeneity of interventions and outcomes researched, a meta-analysis of the data was not possible. Therefore, a narrative synthesis was undertaken to synthesise the quantitative data. Key concepts from the included papers were grouped according to similar interventions, allowing for a clear summary of different results.

## 3 Results

### 3.1 General characteristics

**Fig. 1:**
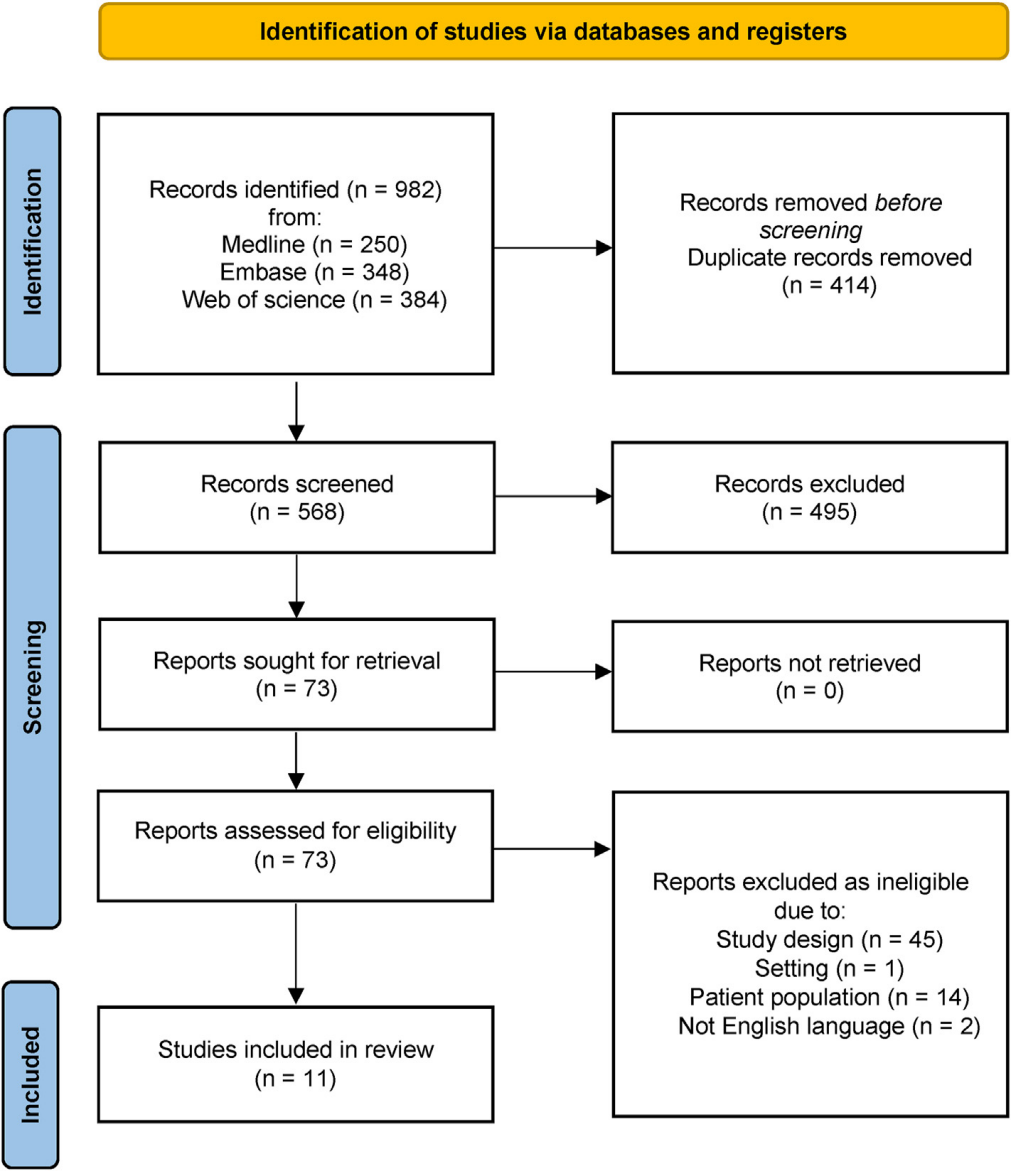
Preferred Reporting Items for Systematic Review and Meta-Analyses flowchart [15].

The initial search strategy identified 982 papers. After manual removal of duplicates and screening of title and abstract, 73 full text articles were screened for eligibility. 62 of these were excluded according to the inclusion and exclusion criteria, leaving 11 studies included in this review. A full breakdown of the search results and reasons for exclusion are found in Fig. 1 [15].

Out of the 11 included studies, one was a retrospective cohort study [22], four were quasi-experimental studies [[23], [24], [25], [26]], and six were randomised controlled trials [5,10,[27], [28], [29], [30]]. Only one of the 11 included studies investigated male athletes, with the other 10 investigating females [10]. A variety of interventions and potential treatments were included (Table 1), including increase in energy intake (n ​= ​3), increase in energy intake and decrease in energy expenditure (n ​= ​1), dietary approaches (n ​= ​1), education and counselling (n ​= ​5) and hormonal intervention (n ​= ​1). The performance levels of the athletes included varied from professional to amateur level. The sporting disciplines also varied, with the most common sport included being running. These are presented in a narrative summary (Table 1).

**Table 1:** Summary of included studies.

Author (year); country	Title	Population	Study design	Population symptoms	Intervention(s)	Key findings
Cobb et al. (2007); USA [30]	The effect of oral contraceptives on bone mass and stress fractures in female runners	Overall, (n = 150) competitive female runners from intercollegiate cross-country teams, post-collegiate running clubs, and road races were included. Of these, (n = 23) withdrew or were lost to follow-up and (n = 42) switched groups, 25.5% of the treatment group discontinued oral contraceptives (OC) after an average of 5.4 months of use, and 38.9% of the control group started taking them at an average of 11.3 months into the study.	Randomised controlled trial	Menstrual disturbance: defined as amenorrhoeic (zero to three cycles in the past year) or oligomenorrheic (four to nine cycles in the past year).	**Hormonal intervention:** 30 μg of ethinyl oestradiol and 0.3 mg of norgestrel to be taken over two years compared to no intervention.	Randomisation to OC was unrelated to changes in bone mineral density (BMD) or bone mineral content (BMC) in those with menstrual status. However, treatment-received analyses showed that runners with menstrual dysfunction who used OC gained approximately 1% per year in spine BMD and whole-body BMC (p < 0.05).
Arends et al. (2012); USA [22]	Restoration of menses with nonpharmacologic therapy in college athletes with menstrual disturbances: A 5-year retrospective study	Charts from (n = 373) Division I female student athletes seen by team physicians over a five year period from University of California, Los Angeles were reviewed. Of these, (n = 51) had menstrual disturbances (14.7% oligomenorrheic, 5.0% amenorrhoeic) and underwent interventions. Out of these, (n = 8) were lost to follow up and (n = 17) were excluded for OC use.	Retrospective cohort	Menstrual disturbance: defined as no menarche by age 15 (primary amenorrhoea), absence of menses for over 90 days (secondary amenorrhoea), menstrual cycles over 36 days (oligomenorrhea).	**Increase in energy intake and decreased expenditure:** Physician counselling and referral to sport dietitian education to increase energy availability (EA) by increasing dietary energy intake and/or decreasing exercise energy expenditure based on individual needs.	17.6% of oligomenorrheic and amenorrhoeic athletes who underwent interventions experienced return of menses (ROM). The mean time for ROM for those with menstrual dysfunction was 15.6 ± 2.6 months. Percent weight gain was a significant positive prediction of ROM (p < 0.05).
Cialdella-Kam et al. (2014); USA [23]	Dietary intervention restored menses in female athletes with Exercise-Associated Menstrual Dysfunction with limited impact on bone and muscle health	Endurance trained females of which (n = 12) had exercise-related menstrual dysfunction (ExMD) and (n = 10) were eumenorrheic controls. Overall, (n = 8) women with ExMD completed the intervention; (n = 4) women dropped out due to personal reasons.	Quasi-experimental	Menstrual disturbance: defined as endurance trained women who were amenorrhoeic or oligomenorrheic.	**Increase in energy intake:Six** month carbohydrate-protein supplement (360kcal/day, 54g carbohydrate/day, 20g protein/day).	Increases in energy intake, availability and balance were not statistically significant between experimental and control groups for any outcome. All ExMD experienced ROM, with a mean time of 2.6 ± 2.2 months for one spontaneous bleed. BMD, muscle power and mood state were not significantly different following the intervention for those in the experimental group.
Keay et al. (2019); UK [10]	Clinical evaluation of education relating to nutrition and skeletal loading in competitive male road cyclists at risk of Relative Energy Deficiency in Sports (RED-S): 6-month randomised controlled trial	Adult male competitive cyclists (n = 50), of which were the equivalent to British Cycling category 2 or above. Overall, (n =5) were lost to follow up so (n = 45) were included in the final analysis.	Randomised controlled trial	At risk of Relative Energy Deficiency in Sport (REDs): as stated in paper.	**Education:** Education relating to nutrition by a registered clinical sports dietitian and skeletal loading exercises over six months.	Results were measured using a questionnaire and clinical interview. Positive changes in behaviour of nutrition and skeletal loading showed a 2.0% increase (p < 0.001) in lumbar spine BMD and negative behaviours showed a 2.7% decrease, both of which were significant.
Stewart et al. (2019); USA [5]	The Female Athlete Body project study: 18-month outcomes in eating disorder symptoms and risk factors	Female collegiate athletes (n = 263) of which were in the experimental group and (n = 218) in the control group. This reduced to (n = 239) in the experimental group and (n = 198) in the control group, after follow up was accounted for.	Randomised controlled trial	Eating disorder symptoms and risk factors.	**Education**: One peer-led 1.3-hr session per week over a total of three weeks. These were small-group interactive sessions focusing on education on REDs, nutrition, sleep, and exercise (to name a few).	Those receiving the intervention showed a significant decrease in dietary restraint in comparison to the control. There were also fewer objective and subjective binge episodes, along with a significantly lower thin-ideal internalisation and increased body mass index in the intervention group (p < 0.05).
De Souza et al. (2021); USA [27]	Randomised controlled trial of the effects of increased energy intake on menstrual recovery in exercising women with menstrual disturbances: the ‘REFUEL’ study	Of (n = 233) women who were screened, (n = 40) ExMD females met the criteria and were included in the experimental group, with (n = 36) in the control group. Overall (n = 17) from the experimental group and (n = 16) from the control completed the intervention.	Randomised controlled trial	Menstrual disturbance: defined as amenorrhoeic (no menses for three months) or oligomenorrhoeic (one or two menses in the past three months or less than seven menses in the past 12 months, or 36 to 89 day cycle).	**Increase in energy intake:** 12 month increased energy intake 20-40% above baseline energy needs for the experimental group compared to maintained energy intake in the control group.	The experimental group was significantly more likely to menstruate during the intervention than the control (p = 0.002). The experimental group had a greater increase in body weight, energy intake and total triiodothyronine compared to the control (p < 0.05). Increasing energy intake led to a significant increase in the likelihood of experiencing menses (p = 0.001). Of those who experienced ROM in the experimental group, 67% did so in three months, 78% in six months and 22% in twelve months.
De Souza et al. (2022); USA [28]	Bone mineral density in response to increased energy intake in exercising women with oligomenorrhea/amenorrhea: the REFUEL randomized controlled trial	Of (n = 233) women who were screened, (n = 40) ExMD females met the criteria and were included in the experimental group, with (n = 36) in the control group. Overall (n = 17) from the experimental group and (n = 16) from the control completed the intervention.	Randomised controlled trial	Menstrual disturbance: defined as amenorrhoeic (no menses for three months) or oligomenorrhoeic (one or two menses in the past three months or less than seven menses in the past 12 months, or 36 to 89 day cycle).	**Increase in energy intake:** 12 month increased energy intake 20-40% above baseline energy needs for the experimental group compared to maintained energy intake in the control group.	There were no significant changes in total body and spine areal BMD (p > 0.05) between the experimental and control group. Both groups experienced a reduction in femoral neck areal BMD at six (p = 0.043) and 12 months (p = 0.023). Both groups experienced a reduction in total hip areal BMD at six months (p = 0.004).
Fahrenholtz et al. (2023); Norway, Sweden, Ireland, Germany [24]	Effects of a 16-Week Digital intervention on sports nutrition knowledge and behavior in female endurance athletes with risk of Relative Energy Deficiency in Sport (REDs)	Female athletes identified at risk of REDs of which (n = 32) were allocated to the experimental group and (n = 18) in the control group. Due to loss from follow up and exclusion criteria, only (n = 15) in the control and (n = 31) in the intervention were included.	Quasi-experimental	At risk of REDs: defined by the Low Energy Availability in Females Questionnaire score of ≥ eight.	**Education and counselling:** weekly lectures in sports nutrition and fortnightly individual athlete-centred nutrition counselling for 16 weeks.	Interviews were used to assess sports nutrition knowledge, for which there was strong evidence to show this improved after the intervention, as well as the self-perceived knowledge. Despite this, there was weak evidence to suggest improvements in sports nutrition behaviours in female endurance athletes with symptoms of REDs.
Fahrenholtz et al. (2023); Norway, Sweden, Ireland, Germany [25]	Short-term effects and long-term changes of FUEL – a digital sports nutrition intervention on REDs related symptoms in female athletes	Female athletes identified at risk of REDs of which (n = 32) were allocated to the experimental group and (n = 18) in the control group. Due to loss from follow up and exclusion criteria, only (n = 15) in the control and (n = 31) in the intervention were included.	Quasi-experimental	At risk of REDs: defined by the Low Energy Availability in Females Questionnaire score of ≥ eight.	**Education and counselling:** weekly lectures in sports nutrition and fortnightly individual athlete-centred nutrition counselling for 16 weeks.	Significant evidence was found for improvements in the Low Energy Availability in Females Questionnaire score and menstrual score among FUEL athletes. Weak evidence was found in gastrointestinal scores. A reduction in Eating Disorder Examination Questionnaire was observed, persisting at 12 months post intervention, suggesting long term benefits on disordered eating.
Fredericson et al. (2023); USA [26]	Healthy Runner Project: a 7-year, multisite nutrition education intervention to reduce bone stress injury incidence in collegiate distance runners	Female middle-distance and long-distance runners from Division I cross-country and track and field programmes. The historical phase included (n = 56) runners and 90.2 person-years; the intervention phase included (n = 78) runners and 137.3 person-years.	Quasi-experimental	Bone stress injury: a symptom of REDs.	**Education:** The intervention ran over four years. Each year, a 15-20 minute team nutrition presentation focused on optimising EA was delivered. 15-30 minute individualised nutrition sessions were also provided, with follow ups depending on the severity of risk of REDs.	Overall bone stress injury rates were not reduced after the intervention when compared to the historical phase. However, there was a significant decrease from 0.18 to 0.10 events per person-year in trabecular bone stress injury (p = 0.047).
Miralles-Amorós et al. (2023); Spain [29]	Study of different personalised dietary plans on eating behaviour, body image and mood in young female professional handball players: A randomised controlled trial	Professional Spanish female handball players (n = 21). None lost to follow up.	Randomised controlled trial	At risk of REDs: players had an energy intake of less than 30 kcal/kg lean body mass per day.	**Dietary approaches:** 12-week personalised diet. Three groups of seven randomly distributed players: the controls followed a free diet with healthy lifestyle recommendations; an experimental Mediterranean diet; and an experimental high-antioxidant diet.	No significant difference was found between groups on eating behaviour, body image or mood. Significant differences in body image, tension, vigour and depression from pre- to post-intervention was found (p < 0.05). Eating behaviour was not statistically significant different.

### 3.2 Critical appraisal

No studies were excluded based on critical appraisal (Table 2, Table 3, Table 4, Supplementary file for table key). However, there were some concerns regarding the rate of dropout in some studies. De Souza et al. [27,28] exhibited dropout rates of 57​%, yet an intention-to-treat analysis was utilised to counteract this. Cobb et al. [30] also found 33​% of participants switched groups and 39​% of the control group began taking OC at an average of 11.3 months, meaning that these results may not be reliable or accurate (Table 2, Table 3, Table 4).

**Table 2:**
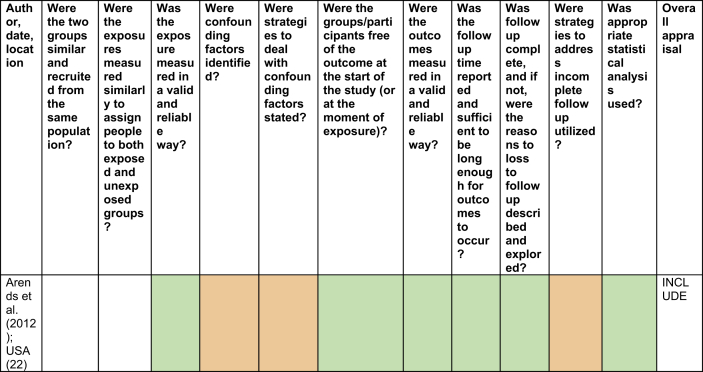
Critical appraisal of retrospective cohort study [12].

**Table 3:**
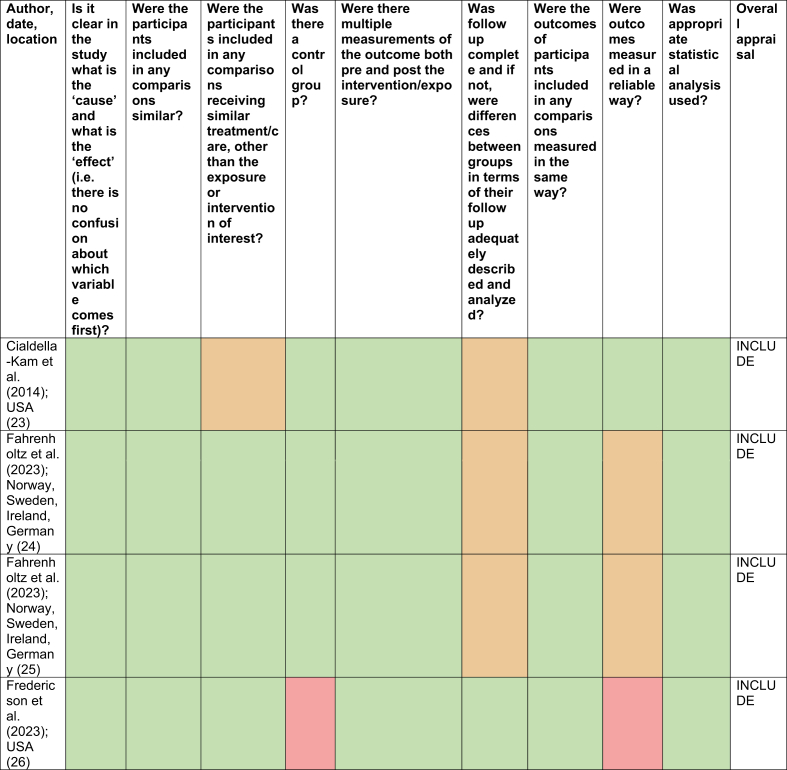
Critical appraisal of quasi-experimental studies [13].

**Table 4:**
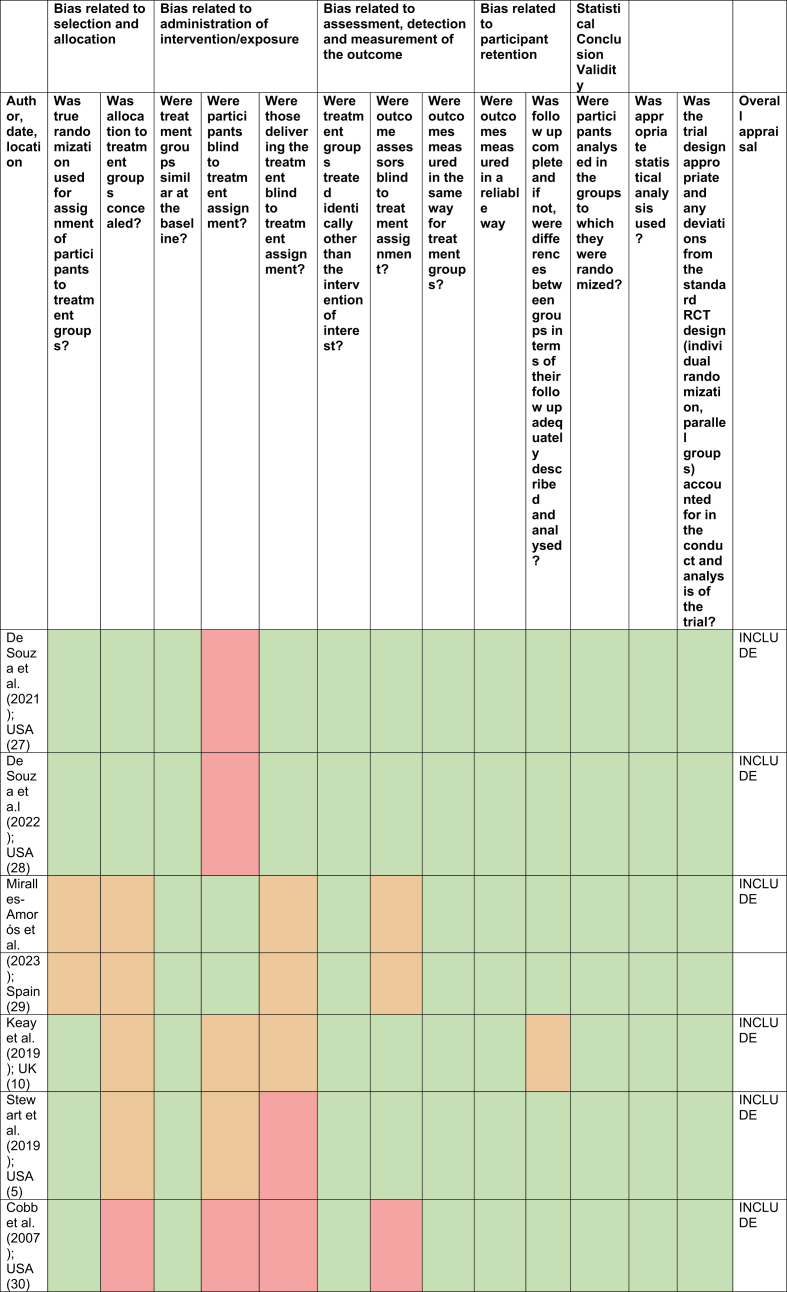
Critical appraisal of randomised control trials [14].

### 3.3 Increase in energy intake and decreased energy expenditure

Overall, three studies investigated the effects of increasing energy intake on athletes experiencing menstrual disturbances [22,23,27]. All three studies showed that increasing energy intake by 250–350 calories or 20–40​% above baseline energy needs could enable return of menses (ROM) [22,23,27]. Time for ROM differed vastly across all studies; Arends et al. [22] found the average ROM to be *M* ​± ​*SEM* 15.6 ​± ​2.6 months, whilst Cialdella et al. [23] reported first menses to occur at *M* ​± ​2.6 ​± ​2.2-months. However, only one study stated that ROM was significant, concluding that increasing energy intake led to a significant increase in ROM, compared to those who maintained energy intake [27]. Two of these studies, by Arends et al. [22] and De Souza et al. [27] showed that ROM was associated with a significant increase in body weight. Cialdella et al. [23] investigated both an increase in energy intake and decrease in energy expenditure and found that all athletes experienced ROM, despite some not experiencing a significantly increased EA after six months.

Two studies investigated the effects of increasing energy intake on bone health, yet neither showed significant changes between the experimental and control groups [23,28]. Cialdella et al. [23] found those with exercise-related menstrual dysfunction (ExMD) which had been present for over eight months, had a lower bone mineral density (BMD) than those with ExMD for less than eight months, yet this was not significantly different [23]. Additionally, De Souza et al. [28] found no significant difference between the experimental and control group for total body and spine areal BMD following increased energy intake, yet both groups experienced a reduction in femoral neck areal BMD at six and 12 months, and a total decrease in femoral neck areal BMD at six months [28]. Cialdella et al. [23] also investigated reproductive and thyroid hormones, muscle power and strength, as well as profile of mood scores. Peak, average and explosive power for ankle or knee flexion and extension did not significantly change following increased energy intake in either group [23]. Peak Profile of Mood scores were not significantly improved following increased energy intake for tension, depression, anger, vigour, fatigue and confusion in those with and without ExMD [23]. Although those with ExMD did exhibit a 15​% lower fatigue score than those without ExMD and depression scores improved by 8​% in those with ExMD following increased energy intake, this was not significantly different [23].

### 3.4 Dietary approaches

Only one study investigated the role of different dietary plans, specifically a free diet (control), Mediterranean diet or high antioxidant diet in professional Spanish female handball players [29]. Miralles-Amorós et al. [29] found that there was no significant differences between the three dietary plans on eating behaviour (measured using the Eating Attitude Test), body image (measured using the Body Shape Questionnaire) and Profile of Mood Scores for fatigue, anger, vigour, depression and tension. However, there were significant improvements within each group for body image, tension, vigour and depression [29].

### 3.5 Education and counselling

#### 3.5.1 Nutrition education

Four studies investigated the impact of a nutrition-based education program as an intervention for REDs [10,[24], [25], [26]]. When dietitian advice on nutrition was combined with skeletal loading exercises, positive changes in behaviours relating to these were seen and significantly increased lumbar spine BMD (2.0​%) in male cyclists [10]. When negative behaviours were seen, there was a significant decrease in lumbar spine BMD (2.7​%) [10]. Moreover, changes in EA were significantly associated with the corresponding direction of sport performance, based on British Cycling points gained over the racing season [10]. Fredrickson et al. [26] showed that a team nutrition presentation every year for four years and individualised nutrition sessions, led to a significant reduction in trabecular-rich bone stress injuries. Fahrenholtz et al. [24] showed that a weekly lecture and fortnightly counselling of athletes over a 16 week period increased knowledge of sports nutrition, self-perceived knowledge, carbohydrate intake, and led to a partially increased energy intake compared to the control group. However, the effects on overall dietary intake and training volume were less clear, with weak or no significant interaction effects [24]. Additionally, training intensity was reduced across both groups without significant differences [24]. At the six and 12 month follow up, there was a 24​% decrease in athletes with a Low Energy Availability in Females Questionnaire score ​≥ ​eight, and therefore at risk of LEA. Menstrual score also improved compared to the control group [25]. The Eating Disorder Examination Questionnaire score also decreased showing longer term benefits of the 16 week intervention [25].

#### 3.5.2 REDs education

Only one study included REDs education as a key component [5]. This was paired with information about other behaviours that contribute to the condition, such as nutrition, sleep and exercise [5]. Three weekly sessions were given in which athletes were encouraged to make behaviour changes related to the information they had been given. The athletes who received this showed significantly reduced dietary restraint, binge episodes and thin-ideal internalisation 18 months after the intervention, compared to those in the control group. These in turn significantly increased body mass index [5].

### 3.6 Hormonal intervention

Only one study in this review focused on hormonal interventions [30]. Cobb et al. [30] concluded that OC did not significantly affect BMD or bone mineral content (BMC) overall, except for a transient reduction in hip BMD in oligomenorrheic women, which was not concluded to be significant [30]. However, athletes who used OC or regained menstrual function both showed approximately 1% increase in whole-body BMC and spine BMD [30]. OC use was also associated with a non-significant reduction in stress fracture risk, yet menstrual status and weight gain were stronger predictors of bone outcomes [30].

## 4 Discussion

This is the first systematic review which synthesised the available literature to determine the efficacy of different interventions in athletes with REDs, to assist in clinical decision making. Three studies found increasing energy intake enabled ROM [22,23,27]. However, increasing energy intake did not significantly increase BMD [23,28]. Dietary approaches significantly improved body image, tension, vigour and depression [29]. Nutrition education improved bone health, EA, and eating behaviours in athletes, while hormonal interventions had limited effects on BMD [5,10,[24], [25], [26],^29^].

### 4.1 Increase in energy intake and decreased energy expenditure

It is important for professionals to understand that interventions for REDs need to be individualised, and that menstrual recovery does not solely equate to the quality of recovery from REDs. In one study, despite the same regime being followed, not all athletes encountered a ROM [22]. Moreover, the length of time for ROM ranged dramatically between studies [22,23,27]. This could relate to the varying definition of ROM used in each study, or there may be an individual susceptibility when considering the efficacy of interventions, and genetics, psychological, neuroendocrine, metabolic aspects need to be considered [22]. From the results, athletes could be advised that increasing EA can induce a single spontaneous bleed within one year, yet regular menses of 36-day cycles or less for over three months can take over a year to return [22].

This review found that restoring EA by increasing caloric intake and decreasing exercise volume in athletes with REDs could be an important intervention. This review suggests that athletes should increase calories by 250–350 ​kcal/day or increase intake by 20–40​% above their baseline energy to enable ROM [22,23,27]. Cialdella-Kam et al. [23] found that despite increasing caloric intake enabling ROM, energy intake was not significantly increased. However, athletes were in and out of a competition season during the intervention, which may have impacted the significance of results.

Increased body weight is a significant positive predictive factor for ROM in those with menstrual disturbances [22,27]. Additional literature also found an association between weight gain and ROM, yet weight gain may result from increased EA, and may not cause ROM [[31], [32], [33], [34]]. As athletes may be cautious of weight gain, it should be made clear that even small weight gains may improve menstrual disturbances [22,23]. Another study supports this claim, concluding that approximately five kilograms of weight gain is associated with menstrual recovery [35].

Moving forward, more long-term randomised controlled trials investigating both dietary and exercise interventions on REDs symptoms are crucial. However, practical and methodological barriers exist; dropout rates of 57​% occurred in the study by De Souza et al. [27] and not all symptoms of REDs, such as restoration of BMD, were improved by increasing EA [23,28]. De Souza et al. [28] attributed the inability to restore BMD to the length of the intervention of only 12 months and mentioned that changes in BMD in those with REDs may be irreversible. Nevertheless, increased energy intake and decreasing energy expenditure is consistent with recommendations from the 2023 IOC consensus on REDs [1].

### 4.2 Dietary approaches

This analysis discovered that different dietary plans may be important for those at risk of REDs. Significant improvements in body image, tension, vigour, and depression were seen following a free diet (control group), Mediterranean diet, and a high antioxidant diet in professional Spanish female handball players [29]. This indicates that such diets could help athletes at risk of REDs. Nevertheless, further research is imperative as this observation is based on one study. Although there was no significant difference between groups for the outcomes measured, studying athletes for longer than 12 weeks may have allowed for significant results [29].

### 4.3 Education and counselling

This review identified that education is an important part of treatment for REDs. Many athletes believe the loss or dysfunction of menstruation, along with other symptoms such as repeated injuries and fatigue, are normal, and may not fully understand the further implications. However, education has not been shown to be an effective treatment in isolation, as knowledge about the condition itself has not been shown to improve behaviours surrounding this, such as an improvement in dietary behaviour [24]. Additional studies carried out showed no change in dietary intake after sports nutrition counselling and life-coaching sessions, resulting in LEA [36]. However, this review showed that nutrition counselling can significantly reduce certain types of bones stress injuries [26] as well as decreasing the risk of LEA [25]. In summary, it is unclear how effective it is to use education solely as treatment for REDs, and ultimately more research needs to be carried out to fully understand its efficacy.

### 4.4 Hormonal interventions

Hormonal contraceptives do not seem to be an effective treatment for REDs, as shown in this review. However, only one study was retrieved, which demonstrated hormonal intervention had only a small positive effect on the incidence of stress fractures in female runners over two years [30]. The menstrual status of athletes (e.g. oligomenorrhoea, amenorrhoea) has been more strongly linked to BMD, despite the use of OC [30]. Therefore, OC could mask the symptoms of REDs, allowing damage to continue without knowledge, such as a loss of BMD. However, as OC can regulate menstruation, runners with menstrual dysfunction who took OC for at least six months gained more spine BMD than those who still showed menstrual dysfunction [30]. On the other hand, previous studies have found that low-dose OC are detrimental to BMD in eumenorrheic athletes [37,38]. Importantly, Nose-Ogura et al. [39] investigated a different form of hormonal intervention, transdermal oestradiol, on BMD in athletes with a low body weight and BMD, showing that those who received the intervention experienced a significant increase in BMD. Similarly, transdermal oestradiol has been shown to increase BMD Z-scores in endurance athletes with menstrual dysfunction, in the spine (2.75​%), femoral neck (5.25​%) and total hip (1.85​%) after 12 months of use, compared to those taking OC or no hormonal intervention [14]. This is supported by current NICE guidelines which recommend hormone replacement therapy (HRT) instead of OC [13]. More research is needed into the potential for OC and HRT to exacerbate or treat REDs.

### 4.5 Strengths, limitations, and future research

A major challenge faced in this search was the lack of primary research into the efficacy of treatment for REDs, with only 11 studies fitting the inclusion criteria. This meant studies had a high degree of heterogeneity and so meta-analysis was not possible, with some categories of interventions only including one study. More high-quality primary research is vital to fully understand their efficacy.

The research found using our search strategy was limited by the age of participants in our exclusion criteria. Only those who were aged 16 years or over were included in this systematic review as this is the age at which the NHS states periods typically become regular [40]. Subsequently, this limited the number of studies retrieved, including important studies on the use of HRT [14,39]. Future research into the efficacy of treatments in adolescents may provide greater insight into REDs. Additionally, with only one of our studies investigating REDs in males, and none investigating para-athletes, research into interventions for these groups is paramount. Alongside this, the importance of treating REDs holistically could be investigated further. There is a strong link between REDs and conditions such as disordered eating, anxiety and depression [5], and therefore psychological interventions such as counselling, support groups and therapy may need to be involved in treatment. This is supported by the IOC consensus which recommends mental health interventions as part of a multidisciplinary approach to REDs treatment [1].

A key limitation within this field of research is the absence of a recognised definition on menstrual recovery. For example, this definition ranged from menstrual cycles less than 36 days for a minimum of three months, to the occurrence of single menses [22,23]. Such ambiguity makes it difficult to determine what constitutes optimal menstrual recovery, and thus success of interventions. Furthermore, amenorrhea and oligomenorrhea are associated with varying hormonal patterns creating uncertainty as to whether definitions can be applied. A global consensus on what best describes menstrual recovery is needed to allow future prospective studies to elicit the time taken for ROM and the extent to which this links to REDs recovery. Furthermore, we were reliant on authors assessment of risk of REDs. For example, two studies included stated the athletes included were at risk of REDs; however, this was not expanded upon [10,26]. Another study stated athletes had oligomenorrhea or amenorrhea, yet they did not include how they defined these terms [23]. Therefore, readers should be aware of potential differences in severity of REDs symptoms between different research papers.

Despite these limitations, this systematic review is the first to include both pharmacological and non-pharmacological interventions for REDs, and the search strategy gathered primary research on athletes from a range of sporting disciplines and performance levels. In this way, the key findings may be generalisable to a wide population of athletes. Moreover, another strength of this systematic review was that a robust double review process was undertaken to minimise bias, and the inclusion of multiple randomised controlled trials allowed for objective conclusions. Nevertheless, many of the interventions had multiple confounding interventions, making it hard to draw conclusions on the efficacy of treatment [10,22]. Alongside resolving critical appraisal issues with confounding variables, future research needs to focus on reducing drop-out rates, yet this may prove difficult in athletes balancing time pressures [27,30]. Although case reports were excluded to ensure studies were of high quality, moving forward, research must utilise standardised methodology to start unravelling the complexities of interventions for REDs.

## 5 Conclusion

The findings of this systematic review inform clinicians, athletes and researchers of the interventions that are most appropriate in the treatment for REDs so that practices can be adjusted if necessary. REDs presents an array of negative implications for both athlete health and performance. Nevertheless, this systematic review indicates that there are interventions available to aid in recovery. Increasing energy intake is essential and can be more effective when used in conjunction with education and hormonal treatment. Despite this, future research is needed that utilises standardised definitions and methodology to enable better analysis into the efficacy of interventions for REDs, in order to inform better clinical practice.

## Funding

This research did not receive any specific grant from funding agencies in the public, commercial, or not-for-profit sectors.

## Declaration of competing interest

The authors declare that they have no known competing financial interests or personal relationships that could have appeared to influence the work reported in this paper.
