# Supplementary material for: Exploring different interventions for Relative Energy Deficiency in Sport (REDs): A systematic review
Source: JSAMS Plus. 2025 Jan 2;5:100085. doi: 10.1016/j.jsampl.2024.100085 (PMC13008464; doi:10.1016/j.jsampl.2024.100085)
Supplement: Multimedia component 1 [file mmc1.docx]

**Supplementary material**

**Search strategy for each database**

1. ‘Relative energy deficiency in sport’ OR ‘female athlete triad’ OR ‘low energy availability’
2. Athlet*
3. ‘treatment’ OR ‘management’ OR ‘intervention’
4. 1 AND 2 AND 3

**Data extraction form**

| Author, publication year and country | Title of article | Study design | Total number of participants, including those who did not complete study | Participant characteristics: Age, sex, sporting discipline, symptoms | Intervention and duration | Outcome | Comments |
| --- | --- | --- | --- | --- | --- | --- | --- |
|  |  |  |  |  |  |  |  |

**Reviewer guidance for data extraction**

| ***Data to be extracted*** | ***Notes for reviewer*** |
| --- | --- |
| Author |  |
| Year of publication |  |
| Title of article |  |
| Country | Country of intervention |
| Study design | Randomised control, experimental etc. |
| Population characteristics | Total number, age, sex, co-morbidity, ethnicity, no. lost to follow up, symptoms or risk of REDs |
| Intervention | Copy and paste text describing intervention  Type of intervention |
| Duration | Give number of months |
| Outcome measured | e.g. bone mass, biomarkers of REDs |
| Comments |  |

**Key for critical appraisal tables**

| Yes |  |
| --- | --- |
| Unclear |  |
| No |  |
| N/A |  |
